# The Fundus Autofluorescence Spectrum of Punctate Inner Choroidopathy

**DOI:** 10.1155/2015/202097

**Published:** 2015-07-22

**Authors:** Miaoling Li, Xiongze Zhang, Feng Wen

**Affiliations:** State Key Laboratory of Ophthalmology, Zhongshan Ophthalmic Center, Sun Yat-sen University, Guangzhou 510060, China

## Abstract

*Purpose*. To investigate the fundus autofluorescence (FAF) spectrum of punctate inner choroidopathy (PIC). *Methods*. This is a retrospective observational case series of 27 consecutive patients with PIC admitted from October 2013 to March 2015, who underwent short-wavelength- (SW-) and near-infrared- (NIR-) FAF imaging, spectral domain optical coherence tomography (SD-OCT), fluorescein angiography (FA), and indocyanine green angiography (ICGA). *Results*. There were three primary findings on the FAF imaging of patients with PIC. First, active PIC lesions revealed hypoautofluorescent spots with hyperautofluorescent margin. After the lesions regressed, the hyperautoflurescent margin faded. Second, subclinical and most of the atrophic PIC lesions appeared to be hypoautofluorescent spots. But subclinical PIC lesions were more distinctive on NIR-FAF imaging than on SW-FAF imaging. Third, hypoautofluorescent spots of PIC lesions coexisted with hyperautofluorescent patches on SW-FAF imaging. These hyperautofluorescent patches were demonstrated to be multiple evanescent white dot syndrome (MEWDS) or acute zonal occult outer retinopathy (AZOOR) lesions by subsequent multimodal imaging and faded during follow-up examinations. *Conclusion*. FAF imaging helps in noninvasively tracking the evolution of PIC lesions and identifying the combined MEWDS or AZOOR lesions, complementary to SD-OCT and angiographic studies.

## 1. Introduction

Punctate inner choroidopathy (PIC), a disease that typically affects young myopic women, is characterized by multiple, small, and yellow-white spots mainly in the posterior pole without signs of anterior uveitis or vitritis. The lesions resolve with time, leaving atrophic spots with variable pigmentation [1].

The PIC lesions are thought to occur at the level of the inner choroid and retinal pigment epithelium (RPE) [1]. Fundus autofluorescence (FAF) imaging includes short-wavelength- (SW-) and near-infrared- (NIR-) FAF imaging; they visualize naturally occurring fluorophores of lipofuscin from RPE and melanin from choroid, respectively [2–4]. Therefore, use of FAF imaging in the diagnosis and follow-up examinations of PIC may help in understanding of the pathophysiology, as well as the interpretation of angiographic and spectral domain optical coherence tomography (SD-OCT) patterns.

In this study, we aimed to analyze the varied FAF manifestations of PIC according to its phases with 27 patients.

## 2. Materials and Methods

27 consecutive patients (35 eyes) with PIC referred to macular disease service of Zhongshan Ophthalmic Center from October 2013 to March 2015 were included in this study. This study's procedures adhered to the tenets of the Declaration of Helsinki and were approved by the Institutional Review Board at the Zhongshan Ophthalmic Center. The diagnosis of PIC was based on the following criteria: (1) presence of multiple, punctate, yellow-white lesions (most ≤ 500 mm) or atrophic scars, primarily in the posterior pole and at the level of the deep retina and choroid; (2) SD-OCT imaging that revealed PIC lesions in any of the 5 stages: choroidal infiltration (stage I), formation of sub-RPE nodules (stage II), chorioretinal nodules (stage III), regression (stage IV), or retinal herniation (stage V) [5]; and (3) no signs of anterior uveitis or vitritis.

FAF images were obtained using a confocal scanning laser ophthalmoscope (cSLO; Spectralis HRA, Heidelberg Engineering, Heidelberg, Germany) in both eyes with dilated pupils. Focus was achieved at 815 nm using infrared reflectance mode. SW-FAF imaging was performed with argon laser light (488 nm) for excitation and a barrier filter allowing for the passage of light > 500 nm. NIR-FAF images were obtained with diode laser light (787 nm) for excitation and a barrier filter allowing light transmission > 800 nm.

Additionally, best corrected visual acuity measurement using Snellen charts, slit-lamp examinations, dilated fundoscopy, fundus photography (Carl Zeiss, Inc., Jena, Germany, or Topcon Corp., Tokyo, Japan), fluorescein angiography (FA) (Spectralis HRA, Heidelberg Engineering, Heidelberg, Germany), and SD-OCT (Spectralis SD-OCT, Heidelberg Engineering, Heidelberg, Germany, or Cirrus HD-OCT5000, Carl Zeiss Meditec, Inc., Dublin, CA) were conducted. Indocyanine angiography (ICGA) (Spectralis HRA, Heidelberg Engineering, Heidelberg, Germany) was performed selectively to evaluate patients with lesions beyond PIC. Two reviewers (Miaoling Li and Xiongze Zhang) assessed all images separately. Discrepancies in their findings were referred to a fundus specialist (Feng Wen) for final determination.

## 3. Results

Of the 27 consecutive patients (35 eyes) with PIC that were included in this study, eight were bilaterally affected. The mean follow-up period for examination was 19.8 ± 18.4 weeks (range, 4–78 weeks).

Three findings on FAF of patients with PIC were observed as follows (Table 1).

### 3.1. Hypoautofluorescent Spot with Hyperautofluorescent Margin

**Figure 1:**
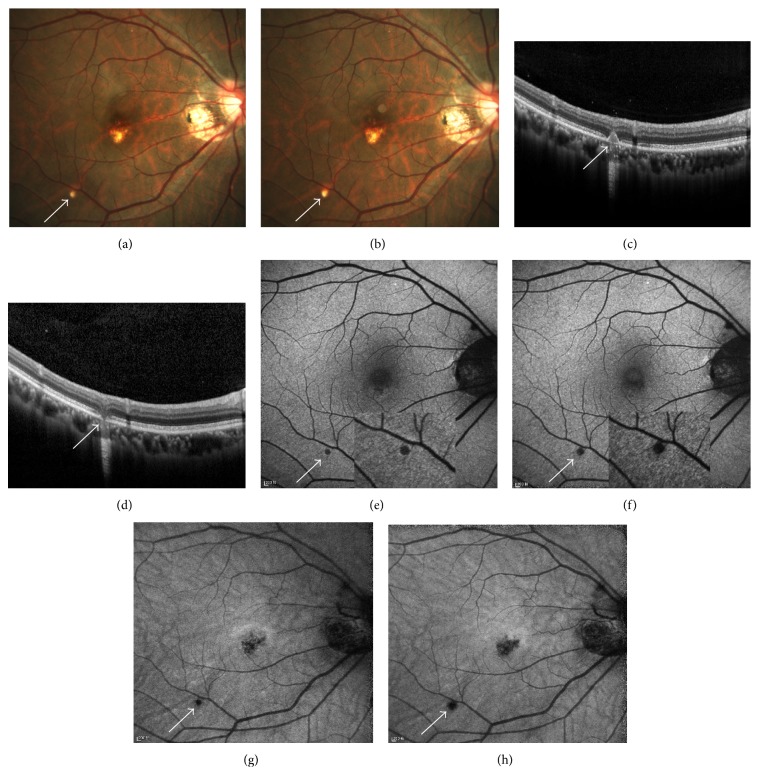
The presence of hypoautofluorescent spot with hyperautofluorescent margin on FAF imaging (e, f, g, and h) and corresponding findings on fundus photography (a, b) and SD-OCT (c, d). (a) A yellow-white creamy spot was shown on fundus photography. (c) SD-OCT revealed a hump-shaped chorioretinal nodule of moderate reflectivity breaking through RPE and Bruch's membrane (BM), beneath the OPL. Hyperautofluorescent margin was detected with SW-FAF imaging (e), while it was not obvious on NIR-FAF (g). Six weeks later, (b) the spot became larger on fundus photography. (d) On SD-OCT, the nodule regressed from the apex toward the choroidal part. (f) The hyperautofluorescent margin on SW-FAF disappeared. (h) The hypoautofluorescent spot on NIR-FAF enlarged.

The hyperautofluorescent margin was more profound when it was imaged by SW-FAF. Active lesions manifested as hypoautofluorescent spots with hyperautofluorescent margin. They corresponded with yellow-white, creamy spots on fundus photography. When imaged by SD-OCT, the spots corresponded with hump-shaped chorioretinal nodules of moderate reflectivity breaking through the RPE and beneath the outer plexiform layer (OPL) (Figure 1). In our previous study, lesions with this characteristic on SD-OCT were categorized into stage III [5]. A small part of atrophic PIC lesions could also be detected as having hyperautofluorescent margin.

### 3.2. Hypoautofluorescent Spot without Hyperautofluorescent Margin

**Figure 2:**
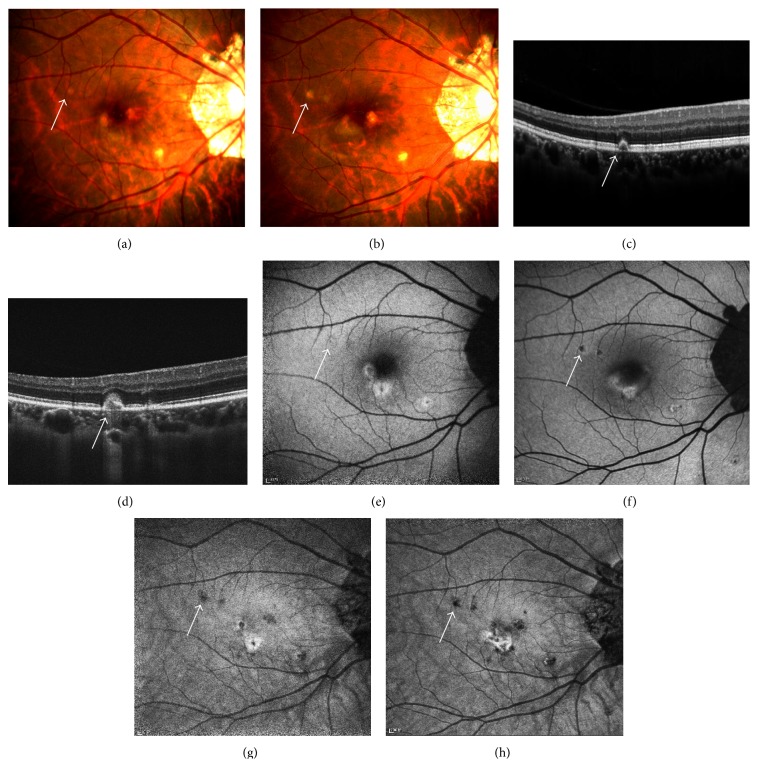
The presence of hypoautofluorescent spot without hyperautofluorescent margin on FAF (e, f, g, and h) and corresponding findings on fundus photography (a, b) and SD-OCT (c, d). (a) A spot with slight discoloration presented on fundus photography. (b) A sub-RPE nodule was shown on SD-OCT, penetrating BM to lift the RPE. A hypoautofluorescent lesionwasmore distinctively shown on NIR-FAF (g) compared to SW-FAF (e). (b) Eleven weeks later, the spot became larger on fundus photography. (d) The chorioretinal nodule on SD-OCT broke through RPE and BM, reaching the OPL. (f) The hypoautofluorescent spot on SW-FAF became more distinctive, with hyperautofluorescent margin around. (h) The hypoautofluorescent spot on NIR-FAF became darker.

There were two situations where PIC lesions appeared to be hypoautofluorescent spots on FAF. First, the majority of atrophic lesions presented as hypoautofluorescent spots. The atrophic lesions can be further divided into stage IV and stage V by fundus examination or SD-OCT based on RPE proliferation, but not by FAF imaging. Second, subclinical spots also appeared to be hypoautofluorescent. And these subclinical hypoautofluorescent spots were more distinctive on NIR-FAF than they were on SW-FAF. They manifested as normal or slight discoloration on fundus examination. Moreover, they correlated with the stage II lesion on SD-OCT, which referred to a focal elevation of the RPE [5] (Figure 2).

### 3.3. Hypoautofluorescent Spot Coexisting with Hyperautofluorescent Patches

**Figure 3:**
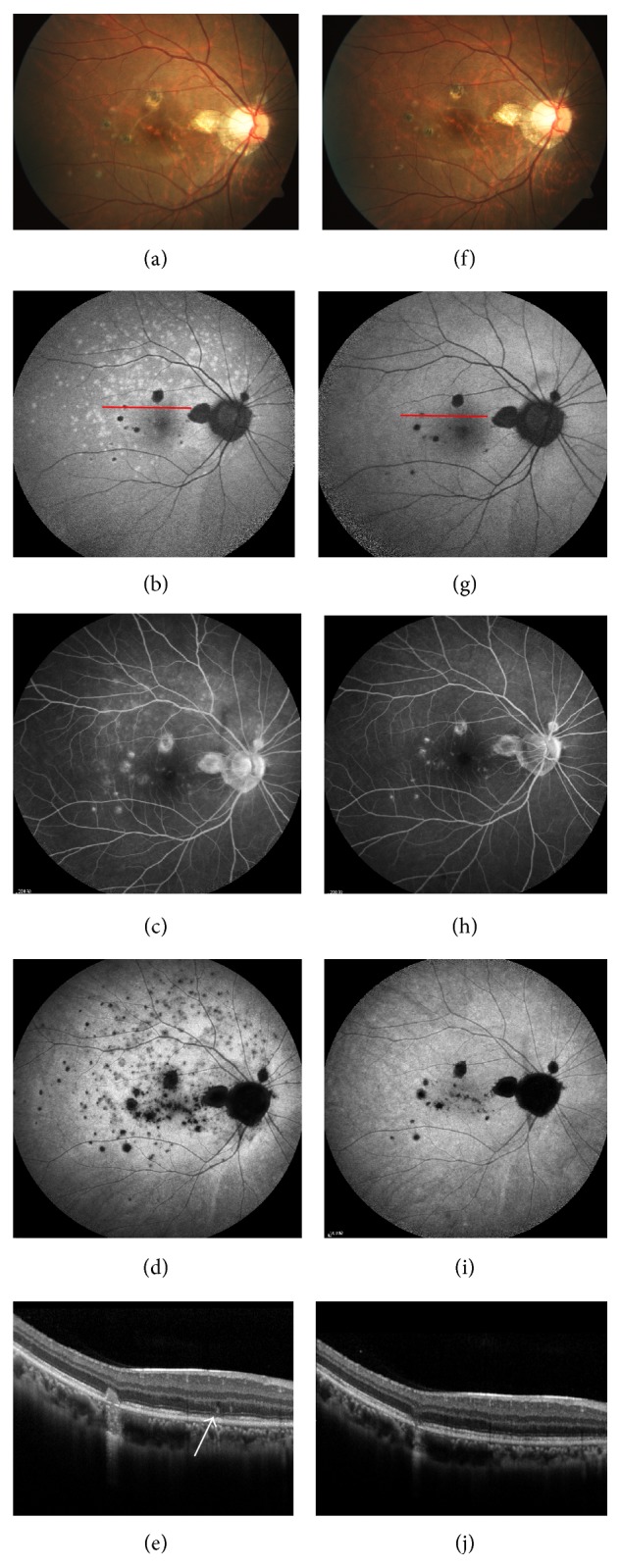
Fundus photography (a, f), SW-FAF (b, g), FA (c, h), ICGA (d, i), and SD-OCT (e, j) of the right eye of a 24-year-old female at the initial visit and 5 weeks later. At the initial visit, her BCVA was 0.2. (a) There were scattered yellowish spots in the upper posterior pole in addition to the yellow-gray PIC lesions. (b) Diffuse hyperautofluorescent areas were shown on SW-FAF, which tended to emerge in the macula and around the optic disc. Additionally, the hypoautofluorescent PIC lesions were surrounded by hyperautofluorescent edge. (c) FA revealed patches of hyperfluorescence, which was especially obvious in late phase and the extent was smaller than it was on SW-FAF and ICGA.The PIC lesions showed hyperfluorescent spots with slight leakage. (d) Hypocyanescent spots were indicated in the late phase of ICGA. (e) The red line (crossing a PIC lesion and hyperautofluorescent areas on SW-FAF) shown on SD-OCT was dome-shaped high reflective materials (arrow), with underlying disrupted EZ. Additionally, an active lesion (stage III) was detected. Five weeks later, the MEWDS lesions faded completely on fundus photography (f), SW-FAF (g), FA (h), and ICGA (i). Meanwhile, the dome-shaped high reflective materials on SDOCT disappeared and disruption of EZ near-completely recovered (j). The hyperautofluorescent margin of the PIC lesion faded on SW-FAF (g). The PIC lesion regressed on SD-OCT (j). Her BCVA was improved to 0.32.

It was detected by SW-AF imaging in 10 of the 27 patients with PIC, with one patient bilaterally affected. All of them had acute visual disturbance, although four were with atrophic PIC lesions. In nine of these patients, the hyperautofluorescent patches were demonstrated to be multiple evanescent white dot syndrome (MEWDS) lesions by multimodal imaging. On fundus examination, the diffuse hyperautofluorescent patches corresponded to scattered yellowish spots but were more numerous. The corresponding areas on SD-OCT manifested as discontinuous of the ellipsoid zone (EZ) with or without dome-shaped high reflective materials. On FA, the retina areas with hyperautofluorescent patches were identified as hyperfluorescence from the initial phase and stained or with slight leakage in the late phase. Using ICGA, they showed multiple hypocyanescent spots during the late phase and were more numerous than the lesions seen under funduscope or by FA. The hyperautofluorescent areas faded completely on follow-up SW-FAF imaging. Meanwhile, the corresponding disruption of EZ recovered and the dome-shaped high reflective materials disappeared on SD-OCT, and the correlated hypocyanescent spots on ICGA became normal (Figure 3). All the patients achieved visual acuity improvement.

**Figure 4:**
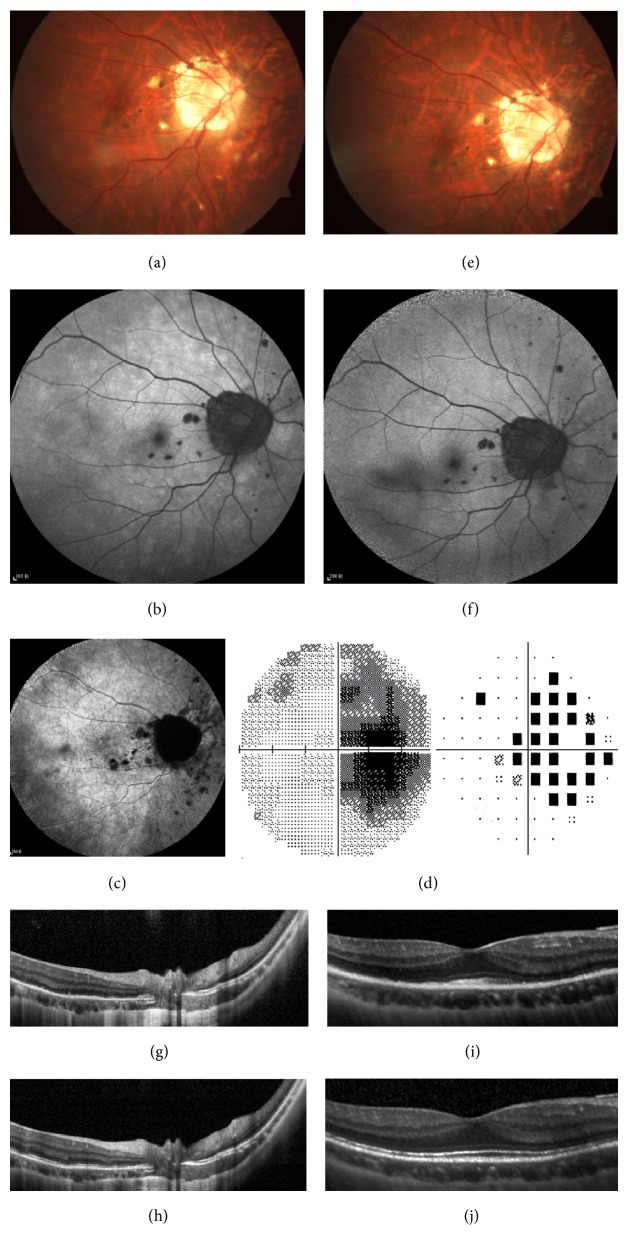
Fundus photography (a, e), SW-FAF (b, f), ICGA (c), perimetry (d), and SD-OCT (g, h, i, and j) of the right eye of a 32-year-old female at the initial visit and 2 months later. The presented BCVA was 0.6. (a) Except for the atrophic lesions of PIC, nothing abnormal was shown on fundus photography. (b) Diffuse hyperautofluorescent patches were detected on SW-FAF, in addition to the hypoautofluorescence of atrophic PIC lesions. (c) Late phase of ICGA was normal in related areas except for some hypocyanescent spots nasal to the optic disc. (d) The visual field examination indicated blind spot enlargement and widespread visual field defects. With relative preservation at the fovea (i), SD-OCT imaging revealed diffuse loss of EZ in the peripapillary zone and in areas that corresponded with the enlarged blind spot. The ONL was preserved (g). Two months later, (e) no obvious change was observed on fundus photography. (f) The intensity of hyperautofluorescence on SW-FAF diminished. (h, j) The EZ reconstructed with focal disruption remained on SD-OCT.

For the remaining one patient, the diffuse hyperautofluorescent patches on SW-FAF were suggested to be acute zonal occult outer retinopathy (AZOOR) lesions by the following examinations. On funduscopic examination, the correlated areas were normal. The electroretinography (ERG) showed decreased rod and maximal responses. The visual field examination indicated blind spot enlargement and widespread visual field defects. Absence of the EZ was detected in the areas of enlarged blind spot and other visual field defects on SD-OCT. There was no abnormality detected in related areas by FA. During the late phase of ICGA, there was no remarkable finding except some hypocyanescent spots nasal to the optic disc (Figure 4). In the follow-up examination two months later, the intensity of hyperautofluorescence revealed on SW-FAF diminished, and the EZ reconstructed with focal disruption remained on SD-OCT (Figure 4). The patient obtained improved visual acuity and visual field.

In this study, subclinical lesions of stage I cannot be detected by FAF imaging.

## 4. Discussion

In this study, different phases of PIC lesions, which are subclinical, active, and atrophic, were distinguished by FAF imaging. Both subclinical lesions and atrophic lesions appeared hypoautofluorescent, but the subclinical lesions were more distinctive on NIR-FAF. Active lesions demonstrated being hypoautofluorescent spots with hyperautofluorescent margin. While some atrophic lesions could also harbor the hyperautofluorescent margin, they cannot be differential from active lesions by FAF imaging independently.

Gass [6] suggested that retinal diseases including MEWDS, multifocal choroiditis and panuveitis (MCP), PIC, acute idiopathic blind spot enlargement (AIBSE), acute macular neuroretinopathy (AMN), and AZOOR should be placed in a single clinical entity called the AZOOR-complex, because they were part of a spectrum of a single disease with similar clinical signs, symptoms, and ophthalmological findings. Moreover, there are several studies which reported that two of these diseases can occur in the same patient either simultaneously or consecutively [7–11]. The appearances of MEWDS on multimodal imaging are as follows: fundus examination reveals multiple, small, yellow-white dots deep to the retina as well as a unique foveal granularity; visual field test reveals blind spots enlargement; FA demonstrates early and late hyperfluorescence of the white dots; multiple small hypocyanescent spots are seen on late-phase ICGA; SW-FAF demonstrates areas of increased autofluorescence that are usually in correspondence with the white dots seen ophthalmoscopically and the site of the focal hypocyanescent spots seen on ICGA; SD-OCT reveals disrupted EZ corresponded with hypocyanescent areas in the late phase of ICGA, with or without dome-shaped high reflective materials; the lesions resolve spontaneously and patients achieve normal vision and visual fields within several weeks to months [12–15]. As compared to MEWDS, the presentations of AZOOR are relatively ill-defined and poorly understood. On initial presentation of AZOOR, the results of fundus examination, FA, ICGA, and FAF could be normal [16]. Abnormal ERG is a critical feature of AZOOR [17]. Blind spot enlargement that is either isolated or associated with one or more other visual field defects is detected by visual field testing [16]. SD-OCT demonstrates irregularity or absence of the EZ, attenuation or loss of the outer nuclear layer (ONL), irregularity of the RPE, and/or decreased retinal thickness. These changes are seen in regions of the retina that corresponded to scotomas [16]. Only a minority of patients experience improvement in visual acuity and/or visual field [17]. Therefore, the diffuse hyperautofluorescent patches coexisting with classic PIC lesions on SW-FAF in our study represented the fact that more than one AZOOR-complex like MEWDS, AZOOR, and PIC had occurred in the same patient.

SW-FAF originates from lipofuscin within the RPE [3]. However, photoreceptors can interfere with SW-FAF imaging recorded by cSLO in two ways. First, under normal conditions, the autofluorescence signal stemming from the RPE is attenuated by absorption of visual pigments contained in the photoreceptors. Second, in the case of loss or damage of photoreceptor cells, RPE lipofuscin levels may vary because of an increased or reduced rate of the shedding of outer segments containing fluorophores [18]. It has been reported that in cases of retinal diseases that affect the photoreceptors prior to RPE the presence of a hyperautofluorescent signal on SW-FAF corresponds to EZ disruption and intact RPE band on SD-OCT [19]. Therefore, the hyperautofluorescent patches may be due to the lack of attenuation of the RPE autofluorescence signal for loss of photoreceptor outer segments or the increased rate of shedding of outer segments, as they manifested as diffuse or multifocal disruptions of EZ with intact RPE on SD-OCT. During the course of the disease, the restoration of the photoreceptor layer, which is likely associated with a reduced rate of shedding of fluorophores or increased absorption, could account for the reduced autofluorescence signal originating from the RPE.

The reasons for the hyperautofluorescent margin of active (stage III) PIC lesions may be the same as it is for the hyperautofluorescent patches. This inference was supported by the gradual elimination of the photoreceptors around the stage III lesions on SD-OCT [5]. As the lesions progressed to the next stage, the surrounding RPE cells became atrophic and experienced a loss of fluorophores. Following this, the hyperautofluorescence at the margin regressed. Nonetheless, the hyperautofluorescent margin around some of the atrophic lesions could have resulted from a window defect through the absence of photoreceptor, indicating progressive atrophy. As evidenced in our previous study [5], the surrounding photoreceptor layer is gradually lost before the atrophy expands. Above all, the hyperautofluorescence of the PIC lesions indicates their progressive nature.

The hypoautofluorescence of subclinical lesions (stage II) recorded by SW-FAF imaging was the result of small clusters of damaged RPE cells overlying focal chorioretinal lesions. As to the atrophic hypoautofluorescent lesions, they were devoid of RPE cells that lacked fluorophores [20].

NIR-FAF comes from melanin in the RPE and choroid, with the latter producing more [3]. Since a stage II lesion is a sub-RPE nodule, the more distinctive appearance of it on NIR-FAF may be due to NIR-FAF's deep layer of examination. For this point, NIR-FAF imaging is superior to SW-FAF imaging at detecting subclinical PIC lesions.

In conclusion, FAF imaging helps in noninvasively monitoring the evolution of PIC lesions, complementary to SD-OCT and angiographic studies. Once diffuse hyperautofluorescent patches are detected in PIC patients, the coexisted MEWDS or AZOOR lesions should be realized.

## Figures and Tables

**Table 1:** FAF and corresponding SD-OCT, FA, and ICGA findings of punctate inner choroidopathy.

FAF		SD-OCT	FA	ICGA
SW-FAF	NIR-FAF			
Hypo-AF spot with hyper-AF margin		Hump-shaped chorioretinal nodule of moderate reflectivity breaking through RPE and BM, beneath the OPL	—	—

Hypo-AF spot		Atrophic lesion: the nodule regresses from the apex toward the choroidal part, with or without RPE proliferation subclinical lesion: sub-RPE nodules penetrating BM to lift the RPE	—	—

Hypo-AF spot coexisting with diffuse hyper-AF patches	Nothing abnormal detected^†^	Diffuse or multifocal disruption of EZ^†^	Normal or patches of hyperfluorescence from the initial phase^†^	Normal or multiple hypocyanescent spots extending from posterior pole to midperiphery on the late phase^†^

BM: Bruch's membrane; EZ: ellipsoid zone; hypo-AF: hypoautofluorescent; hyper-AF: hyperautofluorescent; OPL: outer plexiform layer; RPE: retinal pigment epithelium.

^†^Findings in addition to PIC lesion.
